# Enhancing wireless sensing via a target-mounted intelligent reflecting surface

**DOI:** 10.1093/nsr/nwad150

**Published:** 2023-07-05

**Authors:** Xiaodan Shao, Rui Zhang

**Affiliations:** School of Science and Engineering, Shenzhen Research Institute of Big Data, The Chinese University of Hong Kong, Shenzhen, China; School of Science and Engineering, Shenzhen Research Institute of Big Data, The Chinese University of Hong Kong, Shenzhen, China; Department of Electrical and Computer Engineering, National University of Singapore, Singapore

## Abstract

This perspective paper proposes a new wireless sensing approach by mounting Intelligent Reflecting Surface (IRS) on the target, namely target-mounted IRS, to assist in the detection of the target.

An intelligent reflecting surface (IRS), or its various equivalents such as an reconfigurable intelligent surface (RIS), is an emerging technology to control radio signal propagation in wireless systems. An IRS is a digitally controlled metasurface consisting of a large number of passive reflecting elements, which are connected to a smart controller to enable dynamic adjustments of the amplitude and/or phase of the incident signal on each element independently [1]. Hence, an IRS can generate desired signal reflection patterns and thereby bring forth the vision of smart and programmable signal propagation environments. The core advantages of IRSs stem from their ability to reconfigure the propagation of information-bearing electromagnetic waves in a flexible, low-cost and energy-efficient manner. The functions of the IRS/RIS in boosting wireless communication capacity and reliability have been extensively studied in the literature [2–4].

Driven by recent progress in integrated sensing and communication (ISAC) [5], wireless sensing is expected to become a major service of the next-generation (6G) wireless networks, in addition to wireless communication. Wireless sensing aims to accurately and efficiently detect, estimate and extract useful physical information/features of designated targets by exploiting radio wave transmission, reflection, diffraction and scattering. However, one critical challenge in existing wireless sensing applications lies in the limited target sensing range/accuracy due to the high round-trip sensing signal propagation loss between the radar and the target, as well as the finite radar cross section (RCS) of the target. Motivated by the benefits of an IRS, integrating an IRS into wireless sensing systems provides new opportunities to overcome the above issues efficiently.

Existing IRS-aided sensing studies have mostly considered the IRS as an additional anchor node with known location for improving the sensing accuracy of the target [6]. However, this approach faces a severe issue in practice due to the additional path loss of the sensing signal reflected by the IRS (twice) [7,8], as shown in Fig. 1(a). To overcome this issue of IRS-aided passive sensing, a new IRS active sensing approach was proposed in [9] (see Fig. 1(b)), where the IRS controller sends a sensing signal, and sensors installed on the IRS (namely, semi-passive IRS) receive the echo signal reflected by the target for detection, thus avoiding the sensing signal path loss between the sensing base station (BS) and IRS in conventional cellular sensing systems. However, both IRS-aided passive sensing and IRS active sensing require the IRS to be densely deployed in the network to ensure sensing coverage, which inevitably incurs a higher deployment cost.

**Figure 1.**
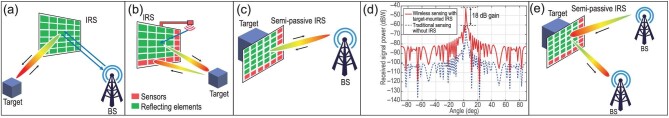
(a) IRS-aided passive sensing. (b) IRS active sensing. (c) Wireless sensing with a target-mounted IRS (monostatic sensing). (d) Received signal power versus BS beam direction. (e) Wireless sensing with a target-mounted IRS (bistatic sensing).

To improve the wireless sensing performance cost-effectively, we propose in this article a new and alternative approach by mounting the IRS on the target, namely target-mounted IRS, to assist in the detection of the target. By dynamically tuning the signal reflection direction of the target-mounted IRS, the angle of departure (AoD) of the reflected sensing signal can be adjusted over time, thereby enhancing the signal power reflected back to the BS for target detection. To this end, sensors (with low-power receive radio-frequency chains, e.g. low-resolution analog-to-digital converter) are installed on the IRS (i.e. semi-passive IRS) to detect the angle of arrival (AoA) of the sensing signal from the BS. In particular, the well-known multiple signal classification (MUSIC) algorithm and estimation of signal parameters via the rotational invariance technique (ESPRIT) can be employed to estimate the AoA from the BS with high resolution. In the context of monostatic sensing, as illustrated in Fig. 1(c), the IRS phase shifts are adjusted based on the acquired AoA, such that the AoD of the reflected signal aligns with its AoA for boosting the signal power received at the BS and hence improving the target detection accuracy. This is particularly useful when there are multi-path signals with different AoAs arriving at the target/IRS, as the IRS can detect the strongest line-of-sight signal’s AoA among them and reflect the signal toward this direction for suppressing the multi-path interference. Note that the received signal power of the proposed sensing scheme scales with the number of reflecting elements *N* to the order of *N*^2^.

To evaluate the performance of the proposed sensing approach with the target-mounted IRS, Fig. 1(d) compares the performance of wireless sensing with a target-mounted IRS and traditional sensing without an IRS versus BS beam direction in a monostatic sensing scenario. In the simulation, we keep the location of the target fixed, while the BS scans different angles of target space via beamforming based on the discrete Fourier transform. The IRS consists of *N* = 128 reflecting elements and eight sensors, while the BS has 128 transmitting antennas and 128 receiving antennas. The distance between the BS and target/IRS is 10 m and it is assumed that a line-of-sight propagation path between them exists. The signal wavelength is λ = 0.2 m and the IRS element separation is *d* = λ/10 [10]. The total physical size of the IRS is *S* = *Nd*^2^ and the target surface covered by the IRS is set as *A* = *S* for ease of comparison. Besides, it is assumed that the target is located at 3° horizontally from the IRS and the RCS of the target is calculated according to 4π*A*^2^/λ^2^. The results show that the received signal power of wireless sensing with a target-mounted IRS gives about 18 dB gain (i.e. 10 log_10_(λ^4^/16π^2^*d*^4^)) over traditional sensing without an IRS at the target angle. This is because, when the BS scanning signal hits the target, the target-mounted IRS can provide a strong passive beamforming gain for the echo signal, which is not available for the target without an IRS.

Besides improving monostatic sensing, the target-mounted IRS can also be used to enhance the sensing performance in bistatic sensing, as shown in Fig. 1(e). Since the AoA and AoD from/to the IRS are different in bistatic sensing, the IRS can first sense the signal directions from/to the transmitting/receiving BSs, respectively, and then set its reflection phases accordingly such that the sensing signal from the transmitting BS can be reflected toward the receiving BS with enhanced power.

Moreover, since the target-mounted IRS can dynamically control the direction of the reflected signal according to its AoA, it is also useful for suppressing the reflected signal power in undesired directions for enhancing the sensing security, e.g. when an illegitimate BS intends to eavesdrop on the echo signal for detecting the target’s direction. It is worth noting that traditional methods of enhancing/suppressing the echo signal by equipping the target with a retrodirective array [11] and absorbing material [12], respectively, cannot control the reflected signal direction flexibly as with our proposed target-mounted IRS.

Research on the target-mounted IRS is still in its infancy, and there are many interesting problems that are open and need to be resolved in future work. In the following, we outline some of the important ones to inspire future research. Firstly, IRS reflection designs for various applications (enhanced/secure sensing) and under different setups (monostatic/bistatic) need to be devised for optimizing the sensing performance. Secondly, in order to extend the sensing range, active reflecting elements can be used to replace the conventional passive reflecting elements, so that an IRS can amplify the echo signal power and simultaneously control its direction. However, the amplification noise introduced by the active IRS reflection needs to be considered in the IRS reflection design. Thirdly, how to exploit the target-mounted IRS to enhance the communication performance of nearby wireless devices is an interesting problem worthy of further investigation, so as to achieve IRS-aided ISAC for enhancing target sensing and wireless communication at the same time.
